# Insects’ Production, Consumption, Policy, and Sustainability: What Have We Learned from the Indigenous Knowledge Systems?

**DOI:** 10.3390/insects12050432

**Published:** 2021-05-11

**Authors:** Letlhogonolo Selaledi, Zahra Hassan, Tlou Grace Manyelo, Monnye Mabelebele

**Affiliations:** 1 Department of Agriculture and Animal Health, College of Agriculture and Environmental Science, University of South Africa, Florida Campus, 28 Pioneer Ave, Florida Park, Roodepoort 1709, South Africa; laselaledi@zoology.up.ac.za (L.S.); zahrabattal@gmail.com (Z.H.); manyelo.t.g@gmail.com (T.G.M.); 2 Department of Zoology and Entomology, Mammal Research Institute, Faculty of Natural and Agri-cultural Sciences, University of Pretoria, Hatfield, Pretoria 0002, South Africa; 3 Department of Agricultural Economics and Animal Production, University of Limpopo, Sovenga 0727, South Africa

**Keywords:** traditional knowledge, entomophagy, mopane, SDG, climate-change

## Abstract

**Simple Summary:**

Insects are the most abundant animals in the world, with all species accounting for more than 70% of the global animal population. To manage the production of insects in the interest of food security, more attention should be given to environmentally-friendly harvesting methods such as indigenous knowledge systems (IKS). Since edible insects have economic, nutritional, and ecological advantages, their production deserves more attention from both national governments and assistance programmes to ensure food security. By developing improved conservation methods, insect production could be readily available throughout the year. Consequently, this review provides information that stakeholders such as farmers and national governments can use to make more informed decisions in relation to the contribution of indigenous knowledge systems to the production and sustainability of edible insects.

**Abstract:**

Edible insects can be produced sustainably, with less environmental impact than other forms of livestock. Globally, over 2000 edible insect species have been reported and are regarded as a great source of nutrition, both as food and feed. Over the years, rural people have used indigenous knowledge to either store or process such insects. However, such valuable knowledge, if not properly recorded and documented, can easily be lost. Thus, there is a need to strike a balance between the use of indigenous and scientific knowledge to produce and process these delicacies. In addition, such indigenous knowledge is vital for preserving biodiversity, since insects are good indicators of climate change as it influences their development, reproduction, and survival. Therefore, successful and sustainable solutions may lie in bringing back indigenous knowledge systems.

## 1. Introduction

Research into using indigenous knowledge systems (IKS) to produce edible insects has been fairly well-documented in certain parts of the world, including the African continent [1,2,3]. However, little attention has been paid to documenting their contribution to the sustainable use of edible insects. While researchers and relevant international organisations, studying the effects of global warming, have shown interest in the sustainable use of resources, the environmental effects thereof have been neglected. Nevertheless, the United Nations (UN) has developed 17 sustainable development goals to guide international societies in achieving a better and more sustainable future for all [4]. However, there have been binary tensions between the contributions of the indigenous knowledge system and the traditional scientific knowledge system, which has led to the romanticisation of the IKS and its decontextualisation [5].

Over the years, there has been an increasing debate concerning how successful indigenous knowledge systems have been in developing effective solutions to the problems facing local people. Many believe that society possesses the tools through which indigenous knowledge can provide the foundation for a generation of new knowledge to address current problems facing society [6]. Subsequently, the belief that indigenous knowledge is not as important as scientific knowledge is rapidly changing [6]. Therefore, it is critical to document this form of knowledge and its contribution to the edible insect sector in light of how it has apparently allowed people to live harmoniously with nature for centuries. Furthermore, indigenous knowledge can aid the design of adaptation strategies to address the complex relationship between phenomena [7]. While the term indigenous knowledge may have different meanings, for the purpose of this study, we use it to refer to “traditional knowledge that has been defined as institutionalised local knowledge that has been built upon and passed on from one generation to the other by word of mouth” [8,9,10]. This knowledge is mainly based on local or customary practices and beliefs and is often difficult to interpret within the context of scientific knowledge [5]. In Africa, an indigenous knowledge system has been applied in various fields such as biodiversity conservation, interpreting climate change, legislation, farming, and in regard to the consumption of edible insects [1,2,3,11].

The use of insects as an alternative source of protein is common in Africa, Latin America, China, Thailand, Japan, and among Australian Aborigines [12]. According to Yen [13], roughly 1500 insect species are consumed in at least 113 countries worldwide. The recent data compiled by Jongema shows that 2111 edible insects species are consumed all over the world [14]. Bangkok, Thailand, is a good example of this since almost 170 species are sold there for food [15]. In China, approximately 324 species, from 11 orders, are consumed [16]. In Australia, the most common species of edible insects are witjuti grubs, Bogong moths, bardi grubs, and honey ants, which are mostly consumed by Australian Aborigines [13]. Itterbeeck and van Huis [17] have reported that in some parts of the world and sub-Saharan Africa, communities that consume edible insects are able to manipulate the natural environment to increase the availability and predictability of such insects. Strategies such as manipulating host tree distribution, shifting cultivation, host tree preservation and manually introducing caterpillars to a selected area have been applied. This has resulted in increased predictability, availability and has improved insect utilisation [17]. These indigenous knowledge methods may have played an important role in addressing issues of biodiversity and conservation of edible insects. A survey conducted by Okia et al. [2] in Rwanda, Uganda, and Burundi revealed that 39 different species of insects are consumed in those countries, with the most common insect species being the katydid grasshopper (*Ruspolia differens*), palm weevil larvae (*Rhynchophorus phoenicis*) and termites (*Macrotermes*). These communities used a system to collect, process and store these delicacies from the wild. However, the study did not highlight the rearing and conservation strategies of the insects consumed by these communities [2]. It is vital that the sustainable consumption of insects be addressed and that mitigation strategies, related to climate change, be developed to prevent these communities from being negatively impacted in the long term. In Tanzania, the edible longhorn grasshopper (*Ruspolia differens*), locally known as *nsenene*, is normally reserved for men, since it is taboo for women and children to consume the *nsenene*. Thus, men have monopolised its consumption. Furthermore, narratives that support certain customs and beliefs—such as pregnant women that consume *nsenene* will give birth to children that resemble a *nsenene* head—are common among local Tanzanian communities [18]. Such practices and belief systems have also been reported in Uganda, where women are prohibited from eating this grasshopper; they are only allowed to catch and cook them for their husbands [18]. The prohibition of women and children from eating this insect could have contributed to the sustainability of the species. However, over time, *nsenene* numbers have decreased and local communities believe that climate change has significantly affected the species [18].

Since Africa is vulnerable to climate change and variability [19], Kenyan farmers, in the Makueni District, who possess indigenous knowledge about rainfall variability indicators, have adapted their practices according to indigenous knowledge-based forecasts [19]. Changes in the weather patterns have influenced the numbers and patterns of emergence of insects during the onset of the rainy season [20]. Climate change is going to affect all living organisms and insects are not an exception [20]. In Africa, sun-drying is a common processing method of edible insects after collecting them from the wild [21]. African temperatures, in recent years, have been warming at a rate that is comparable to that of other regions, while even faster than global mean surface temperature. Therefore, a reduction in precipitation and increasing temperatures could have adverse effects on the African continent. Consequently, these changes threaten socio-economic development, human health and food security [22]. In recent years, the African continent has faced several events that include the invasion of the desert locust, devastating floods and droughts. Therefore, the incorporation of science-based climate information and indigenous knowledge systems could act as a basis for successful climate change adaptation. Community-based adaptation to climate change is one of the methods that indicate that collaboration between scientific and traditional knowledge system could be engaged to effectively address the impact of climate change on societies [23]. This process could be seen as enriching science with traditional knowledge. Furthermore, insects produce less carbon emission of greenhouse gases in the atmosphere and that could benefit the environment and sustainable development [24,25]. However, there is a lack of well-defined legislation on the production of edible insects in countries where entomophagy is practised, which hinders the development of the edible insect’s sector [25,26]. Most of the studies on edible insects are mainly on nutritional composition, processing techniques, consumer acceptance, and entomophagy [27,28,29]. Despite a plethora of literature on edible insects, studies on the contribution of indigenous knowledge to the production and sustainable use of edible insects are still limited. Consequently, this review will discuss the importance of the indigenous knowledge by addressing the following topics: how it has contributed to the sustainable use of edible insects; the need to understand conservation methods used by communities that consume edible insects; and how edible insects can contribute to sustainable development goals and emphasise the importance of developing the legislative framework for the use of edible insects.

## 2. Methodology

This review mainly focuses on the contribution of indigenous knowledge on the production and sustainability of edible insects. The review process was undertaken using articles from various databases using peer-reviewed journals, books, and conference proceedings published. The search was done using the following range of keywords and phrases in each database: indigenous knowledge, sustainability, edible insects production. This was done analytically by focusing attention on a mixture of the words and phrases, therefore, authors searched in databases such as Scopus, Web of Science, Science Direct, and Google Scholar. In the first step of elimination, the search process was conducted using the Google Scholar search engine, where 25,100 studies were listed when indigenous knowledge systems, sustainability, edible insects production were used. The overwhelming majority of these references were evaluated as irrelevant from the point of view of our study. The search was narrowed down by using a timescale of from 2000 until 2021 in order to be able to have wide information about new and old studies in completely indigenous knowledge systems, and 17,800 references were obtained. After the elimination, a final 127 remaining full-text studies were consequently assessed for eligibility.

## 3. Description and Distribution of Edible Insects

Edible insects are known to have adequate protein and a high content of unsaturated fatty acids and minerals like iron and zinc [28]. In Africa and other regions of the world, edible insects such as mopane worms, edible stinkbug, grasshoppers, palm weevil larvae, yellow mealworm, black soldier fly, house fly maggots, silkworms are commonly consumed. Black soldier fly (*Hermetia illucens)* and yellow mealworm (*Tenebrio molitor*) are the most promising species of edible insects [30]. In this section, there follows a brief description of these insects. There is a strong link between the use of these insects and indigenous knowledge systems [18,31,32,33].

### 3.1. Mopane Worms (Gonimbrasia belina)

Mopane worms are edible insects that originate from the warmer parts of Southern Africa and are normally common in semi-deserts, the bushveld, and grassland. They are also found in most parts of Africa such as Angola, Botswana, the Democratic Republic of Congo (DRC), Malawi, Mozambique, Namibia, Southern Zimbabwe and Zambia. Their distribution is closely related to one particular type of mopane tree (*Colophospermum mopane*) and their habitats spread over approximately 384,000 km^2^ of forest [34]. Mopane worms are characterised by having a black colour, with round scales, with a whitish-green and yellow band covered with fine white hairs [35]. Similar to most other edible insects, their life cycle starts when they hatch in the summer. After hatching, the caterpillars will start feeding on the foliage in their immediate environment and move to the next area as soon as they have finished all the foliage [36]. Normally, the larvae grow and moult about four times in different larval stages, after which they are considered as mature mopane worms that can be harvested [35]. These worms are known to be bivoltine, which produces two generations between November and January, and March and May [37].

### 3.2. Edible Stinkbug (Encosternum delegorguei)

The edible stinkbug (*Encosternum delegorguei*) originates from southern and central Africa. In time, the species has spread to rural areas of the DRC, Malawi, South Africa, and Zimbabwe [38,39]. In addition, this species is also widely distributed within the tropical/subtropical woodlands and bushveld of South Africa, Botswana, Swaziland, Malawi, Mozambique, and Namibia. More specifically, research has recorded abundant South African populations in most parts of Bushbuckridge, Ga-Modjadji, and Thohoyandou. The edible stinkbug is known to be highly nutritious, with proteins rich in essential amino acids [40]. Edible stinkbugs have shield-shaped and flattened bodies, averaging 25 mm in length, with small triangular heads, while they appear to be light green-yellow in colour [41]. The stinkbug has forewings that are hardened at the base and membranous at the tips; the hardened bases are separated by a triangular scutellum.

### 3.3. Palm Weevil Larva (Rhynchophorus ferrugineus)

Palm weevil larvae are edible insects originating from Southeast Asia and are found in most parts of India, Africa, Europe, and the United States [42]. They have a very large body and are very long in length, with a rostrum of about 35 to 40 mm. According to Giblin-Davis et al. [43], they have a long nose, which is normally used by female beetles to penetrate a palm tree trunk, to create access wounds in which they deposit their eggs. While this insect varies in colour, which has led to their erroneous classification, most adults are reddish-brownish in colour, full-grown larvae in the trunk are 5 cm long, cone-shaped, cream-coloured, and have no legs [42].

### 3.4. Yellow Mealworms (Tenebrio molitor L.)

The yellow mealworm (*Tenebrio molitor*) is an edible insect indigenous to Europe, but which can now be found worldwide. They come from the *Tenebrionidae* family of two species of darkling beetles of the yellow mealworm beetle and the smaller and less common dark or mini mealworm beetle [44]. The yellow mealworm develops through four life stages, namely egg, larva, pupa, and adult stages. While the larvae typically measure about 2.5 cm or more, the adults are generally between 1.25 and 1.8 cm in length additionally, this species normally overwinters in the larval stage and pupation occurs in spring and early summer, at which time the adult worm will begin to appear [45]. The yellow mealworm is a worm-like larva with a hard exoskeleton. Its body is designed to burrow, eat, and store fat. Like most edible insects, the yellow mealworm also has three distinct sections, which are the head, thorax, and abdomen [46]. These insects are easily bred and fed because they are rich in proteins with a good amino acids profile. For these reasons, they are being used as protein feed for animals such as birds, reptiles, small mammals, and fish. According to Aguilar-Miranda et al. [47], yellow mealworms are usually fed live, but can sometimes be canned or dried, or sold in powder form [43,48].

### 3.5. Black Soldier Fly (Hermetia illucens)

The black soldier fly (*Hermetia illucens*) belongs to the *Stratiomyidae* family. It originated in the tropical, subtropical, and warm temperate zones of America, but was later distributed to many other parts of the globe, especially tropical and warmer temperate regions [49]. When conditions are good, the larvae mature in two months. However, if there is not enough food, this stage can last up to four months [48]. The matured larva is about 27 mm in length and 6 mm in width, while an adult can grow up to 15–20 mm in length [48]. Black soldier fly larvae appear dull and whitish in colour. Normally, one larva can be fed from 25 to 500 mg of fresh matter, with a mix of decaying organic materials such as rotting fruits and vegetables, and grains [49,50].

### 3.6. Silkworms (Bombyx mori)

Silkworms are considered a delicacy in certain parts of the world such as in China, Japan, Thailand, and India [34,51]. Silkworm pupae meal has been found suitable as a livestock feed for monogastric species (poultry, pigs and fish), and also for ruminants [52], due to its high protein content. Silkworms are the caterpillars of moth species bred for silk [53,54]. When it enters its pupa phase, it builds a protective cocoon made of raw silk. At the end of pupation, freshly spent silkworm pupae spoil rapidly, due to their high water content; therefore, spent pupae are generally sun-dried and ground [54,55]. However, they are sometimes killed by boiling, drying or soaking them in NaOH to produce silk [54]. Spent worms are regarded as waste material and are normally discarded in an open environment [56]. According to Blair [57], silkworms have a high fat content, if defatted, silkworm pupae meal is less perishable and results in high protein content. 

### 3.7. Grasshoppers (Caelifera)

Grasshoppers are edible, large-sized insects with a length of about 72 mm [58]. They are found in parts of Africa and North America [59]. Grasshoppers are known to be univoltine, with a few species producing two generations per year, in warmer habitats, and some alpine species requiring two years to complete a single generation [60]. They lay eggs mainly in winter in the soil, although a few species hatch in late August. Hatching takes place from early May to mid-August, with a hatching window of about 2 to 4 weeks. After hatching, a grasshopper nymph passes through four to five instars, with each stage lasting 4 to 7 days, depending on the species and the temperature. Adult females typically have a 1- to 2-week pre-oviposition period, during which they mate. Their eggs develop internally. The movement of grasshoppers is a function of both walking, hopping, and flight [59]. 

## 4. Effects of Climate Change on Edible Insects

Global warming is adversely affecting the earth’s climate and its profound effects are seen in virtually all ecosystems. Every living animal will be affected in one way or another by climatic changes and insects, being an integral biotic component of nearly all ecosystems, are not exempt [20]. Several factors of climate change have been identified as influencing the reproduction of insects. For instance, weather conditions, precipitated by climate change, favour the increased emergence of insects [61,62,63,64,65,66], since moisture and temperature play a significant role in insect ecology. Furthermore, climate changes affect insect population by influencing benthic fauna and the biodiversity that sustain them. Moreover, the unpredicted seasonal changes could affect the temporal distribution of the emergence and the number of insects. Insects have been reported to be sensitive to temperature increases and are known to be more active in higher temperatures [61,62,63]. Several studies have reported that, if climate change involves global warming, there will be more insects. Indeed, the observed increase in temperatures with the current climate change has favoured increased reproduction of the many other insects, as observed by Dunn and Crutchfield [61].

Local knowledge is vital for preserving biodiversity, since insects are good indicators of climate change. This is because it influences their development, reproduction, and survival [67]. Food security, in the face of climate change, is a reality that needs urgent attention; subsequently, in comparison to conventional livestock, the breeding of edible insects may have some environmental benefits [17]. In this regard, Elia et al. [68] have reported that villagers in Tanzania use insects, in particular, to predict the weather. The appearance of millipedes, grass-green grasshoppers and butterflies in large numbers is a sign that rain is imminent. More specifically, the appearance of the Mbilazi insect (a local insect in Tanzania) signifies the amount of prospective rainfall, which can be determined according to the colour of the insect—green means there will be heavy rainfall, while red indicates less. It was further reported that termites can also be used to predict rainfall; their appearance on the ground indicates the beginning of the rainy season. Furthermore, the appearance of and the noise made by the insects in Tanzania are vital indicators of upcoming weather, which assist local communities. Such environmental changes could have an impact on the reliability and usefulness of knowledge held by villagers and farmers in the specific Tanzanian villages of Malunga and Chibelela [68].

## 5. Traditional Knowledge Regarding the Production and Processing of Edible Insects

The knowledge of indigenous practices can contribute to the sustainable development of edible insect sectors. The rearing and processing methods are key to the preservation of edible insects; if done well, they can contribute to improving shelf life, eliminating pathogens and reducing the number of toxic phytochemicals found on insects [20]. The common, traditional method of processing edible insects, after harvesting from the wild or rearing, are species-specific methods such as boiling, sun-drying, toasting or frying. By adding salt, pure honey and palm oil additives, it is possible to improve the shelf life of harvested insects [12,21]. However, sanitary issues have been raised about the traditional processing methods of edible species [12]. Concerns such as unhygienic processing environments, food safety, and food-borne diseases have been raised in the past [12].

While the African continent has over 470 species of edible insects [69], most are harvested from the wild [66]. Furthermore, there are differences in preferences and collection methods of edible insects among various ethnic groups [70]. Therefore, the breeding and processing of edible insects are of great importance. In light of this, the European Food Safety Authority [71] has indicated that the risks associated with the production and processing of insects, as well as relevant environmental factors, must be taken into consideration when production and processing strategies are developed. Schabel [72] has further supported this assertion. These particular challenges are the reason that potential consumers are still sceptical about insects as food and feed. Furthermore, the European Food Safety Authority gave an opinion on dried yellow mealworm (*Tenebrio molitor* larva) as a novel food, the panel concluded that the novel food yellow mealworm is safe to be used whole, dried insect in the form of snacks, and as a food ingredient in a number of food products [73]. In Tanzania, the commercialisation of *nsenene* has improved the collection methods and the technologies used in this process—traps made of iron sheet, bright light bulbs, and large buckets are used to capture the insects at night [18]. However, in order to establish the sustainable, economically feasible mass breeding of edible insects, local people or indigenous groups must be included in such projects [13].

According to Mozhui et al. [74], the Naga tribe, in Northeast India, possess traditional knowledge on how to process edible insects. The knowledge that is utilised relates to the amount of water used to boil the insects, which spices or local ingredients to use, and how to roast certain insects. They are also knowledgeable about the local ingredients used to enhance flavour, including ginger, garlic, dried/fermented bamboo shoot, and murraya powdered fruit, and know the stage and season in which it is best to capture and consume certain edible insects. For instance, with regard to dragonflies (*Anisoptera*), the nymphs are preferred, which are mainly collected during the winter season. The nymphs are then boiled in a small amount of water and cooked until dry. Then, the adult dragonfly wings are removed, fried and eaten as snacks. This knowledge is not only limited to the Odonata (dragonfly belong to Odonata), it extends to the Orthoptera. Grasshoppers are normally sun-dried and preserved for future use and common methods to prepare them include cooking and frying, and the use of local spices. They also consume katydids, which are roasted and eaten as snacks, and *Elinaea securigera,* locally known as Anishe, is an important delicacy of the Naga tribe and is regarded as health food. Field crickets are dug up after pouring water into their burrows, while other crickets are light trapped and collected at night. Sometimes, the Naga tribe use wheat bran to trap crickets, using nets, or they would handpick them and the collected crickets are either cooked, fried or boiled. Certain ethnic groups prefer to flavour the crickets with salt and would prepare them in the following manner: bamboo shoots are put inside 30 cm bamboo pipes and banana leaves are used to close the pipe and to enhance the flavour; then, the bamboo pipes are then placed on a fire, for approximately 25 min, before the dish can be served. The Naga tribe’s rich traditional knowledge of edible insects can be further extended to the Mantodea and Hemiptera. Insects such as mantises are regarded as a delicacy and preferred as snacks. After collection, the head and digestive tract of mantises are removed before drying. The stinkbug is preferred in their adult stage, even though the pungent smell is disliked. However, it has also been reported that the nymphs are tastier than the adult [74]. In order to support the sustainable use of such insects, the Naga tribe make constant efforts to avoid overexploitation, which include practices such as leaving the queen, along with some workers, during the harvesting of bees and wasps to allow development and production.

The rich cultural heritage of the people of the north-eastern region of India is not limited to capturing edible insects from the wild; they have also developed the methodologies to domesticate insects like silkworm. The traditional method used to rear silkworms in Northeast India can be grouped into categories, such as methodologies or technologies used before rearing and methodologies and during-rearing technologies. The use of fresh cow dung, to protect the egg host plant from grazing animals, is common among the producers, while the use of banana leaves, to spread the eggs during the incubation period, is common among *Muga* breeders [75]. Sanitary control measures against secondary infections are widely applied, such as the hanging of the equipment used during the rearing phase above the kitchen fire and restricting the entry to the fields during the breeding stage. These are some of the holistic approaches that silkworm producers have adhered to, to ensure a better crop of *Muga* silkworm [75]. Moreover, this approach has had an enormous impact on the livelihood of the local economies and biodiversity conservation as well as knowledge sharing and transfer within the local populace. Therefore, utilisation of the indigenous knowledge system and the development of better technologies will help to sustain this sector and the local communities that depend on it [75]. In Latin America, countries such as Brazil, Colombia, Ecuador, Mexico, Peru, and Venezuela have a strong history of consuming insects. Eggs, larvae, pupae, nymphs, in some cases adults, are part of the traditional dish of local communities. Entomophagy in Latin America is not only focusing on the nutritional qualities of the insects, some insects are believed to have medicinal purposes [76]. The intergenerational knowledge could have contributed to the sustainable exploitation of these renewable resources. In some rural communities of Mexico, insects provide important economic and nutritional value. The communities are well-skilled in identifying edible insects, collecting, toasting, and preserving them [77].

In South Africa, specifically, knowledge of edible insects is not well-documented [25]. This could be due to urbanisation and a preference for Western cuisines [78,79]. In Southern Africa, indigenous knowledge about the mopane worm, which has been consumed and traded since prehistoric times, plays a major role in sustaining the production and consumption of this insect. For example, the knowledge about the collection, processing and consumption, as well as where and when to collect the insects, is deeply entrenched within local communities [80]. The mopane worm collectors in Botswana are aware of the relationship between the insect’s continued existence and environmental factors such as fluctuations in rainfall that influence the availability and abundance of this particular worm [80]. As the consumption of insects witnesses a decline, solutions must be found to resurrect it as a source of food security. Successful and sustainable solutions lie in reintroducing the indigenous knowledge systems [81]. Table 1 summarises the production and processing method of common edible insects in Africa.

In Australia, the Aborigines have long understood the importance of entomophagy as a sustainable source of food; they had a clear understanding of the danger of overexploitation. The common insects consumed by the Australian Aborigines are witjuti and bardi grubs, Bosong moths, and honey ants [84]. While entomophagy is accepted as a normal part of the diet in many continents, a phobia of eating insects is common in Western societies, therefore, the following section discusses the consumers’ attitudes towards entomophagy.

## 6. Consumers’ Attitude towards the Use of Insects as Food or Feed

Current food production systems have an impact on the environment [82,83]. Edible insects are among the environmentally-friendly sources of proteins [85]. The adoption of insects as food or feed, if they can be produced in sustainable quantities, can increase consumer acceptance of insects as an alternative source of protein [86]; for instance, free-range chickens consume insects such as termites, grasshoppers, and many others. The integration of insects as poultry feed in commercial diets is still being adopted and this source of protein has not yet been fully integrated into commercial feeds [86]. Edible insects are being promoted as an alternative source of protein; however, the major challenge will be creating sustainable production systems that will safeguard the environment and ensure food safety and security [13]. For the environmental, social, and economic benefits of edible insects to be realised, the general attitude of consumers towards edible insects must change in order to increase the rate of adoption [87]. Even though the culture of eating insects is still common in Africa, Asia, and Latin America, there are people that see this practice as barbaric and disgusting [83]. According to Hlongwane et al. [1], in the South African province of Kwazulu-Natal, only 28% of respondents still consume insects compared to 95% of respondents from the province of Limpopo. This highlights a decline in the consumption of insects in certain parts of the country. Furthermore, in Korea, one can purchase canned silkworm pupae at formal markets as snacks [88]. A study conducted by Ghosh et al. [89] reported that Koreans were more prepared to accept insects as food compared to Ethiopian respondents; this could be influenced by many factors such as culture, tradition, history, and familiarity. Edible insects are traditionally preferred because of their availability, size, taste, palatability, and market and ethnomedicinal knowledge [90]. However, in Western countries, entomophagy is not widely accepted even though, certain European countries such as the Netherlands, Belgium, United Kingdom, and France allow a number of insect species to be used as food, and have created some legislative framework to allow for this [91]. However, the tolerance policy that existed in Belgium was abolished and replaced by the application of the transitional measures [92]. The transitional measures stipulate that foods that do not fall within the scope of Regulation (EC) No 258/97 but are now considered novel food may continue to be marketed until a decision has been taken regarding their endorsement as a novel food. This can only be done under the condition that those foodstuffs were legally placed on the market before the 1st of January 2018 and an application for authorisation as a novel food was submitted by the 1st of January 2019. Therefore, the following insects meet the criteria of the transitional period; house cricket (*Acheta domesticus*), lesser mealworm (*Alphitobius diaperinus*), banded cricket (*Glyllodes sigillatus*), African migratory grasshopper (*Locusta migratoria),* and yellow mealworm (*Tenebrio molitor*) [92].

According to Manditsera et al. [93], 63.9% of respondents in rural Zimbabwe consume insects three times per week, compared to 14.5% of urban respondents. The urban respondents reported that the medicinal properties and nutritional value of insects are key elements that lead them to eat insects. It was further reported that insect consumption was negatively related to educational background and monthly incomes of the urban respondent; however, the socio-demographic of rural respondents did not relate to the consumption of edible insects. The development of insect rearing farms and insect value chains using traditional knowledge systems could ensure the sustainable use and promotion of entomophagy (consumption of insects). The consumption of insects, as food and feed, is usually promoted for three main reasons, namely nutritional value, improvement of socio-economic factors, and environmental benefits [94,95]. Educating consumers about the nutritional, ecological and cultural benefits associated with entomophagy is likely to reduce this food neophobia [96]. A study conducted by Orsi et al. [97] reported that only a few German participants were willing to try insects, and it was suggested that the most promising strategy to encourage consumption was to use a processed insect instead of a whole insect. Furthermore, Sogari et al. [30] recommend that further studies should focus on consumer’s point of view more, especially on sensory perception and willingness to pay for animal products fed insects.

## 7. Edible Insects’ Role in the Attainment of the Sustainable Development Goals (SDG)

Indigenous knowledge is a valuable resource that could contribute to the high efficiency and sustainability of the development programme [98]. The indigenous knowledge system with regard to food processing practices forms the bedrock of a community’s composite and collective wisdom, which is passed down through each generation. Brokensha et al. [99] have observed that the incorporation of indigenous knowledge into development planning is a crucial step in any successful development; it is also considered a courtesy to the people concerned. The success of any development programme needs to take into consideration the indigenous knowledge of the local people to effectively ensure sustainability of the development projects [100]. The Food and Agriculture Organization (FAO) of the United Nations hosted a conference, in 2014 and 2018, that was entitled, “Insects to Feed the World”, to deliberate on the importance of insects as food and feed. It was determined that insects are considered a reliable source of energy, protein, and fats [101]; therefore, it makes sense that they can play a positive role in both human and animal nutrition. As already highlighted, the nutritional composition of insects indicates that it is high in amino acids and monounsaturated fatty acids, which meet the requirements of humans [101]. They are also rich in certain vitamins and minerals [102]. In addition, they offer a cheap and efficient way to improve the lives of vulnerable and poor communities and the nutritional value of those that keep to traditional diets, since protein is required in order to meet the goal of adequate nutrition; however, the conventional sources of protein are expensive and, in some cases, not readily available. Insects can fill the gap, as they can be a dietary alternative, which can help to achieve some of the goals with regard to the daily protein requirements. The use of complementary products, containing insect ingredients such as termites and lake flies has played an important part in Kenya’s ability to combat child malnutrition [20]. In South Africa, the mopane worm plays an important role in the local economies [103]. Finke et al. [104] have reported that the consumption of 100 g of black soldier fly larva can assist in meeting the recommended iodine intake of adults. In Southern Africa, these famous worms (*Imbrasia Belina*), the mopane worm, contribute to both rural and urban households as a valuable source of protein [105]. In light of the abovementioned examples, edible insects should be viewed as part and parcel of sustainability and food security for a rapidly growing human population.

Furthermore, Dicke [106] states that insect products can offer positive effects in terms of the maintenance of the health and welfare of livestock, with potential to reduce antibiotic use in livestock production. It is argued that the conventional protein supply is linked to biodiversity loss, nitrogen, and carbon emission acceleration, leading to climate change [107]. However, although edible insects have less of a negative influence on the environment, the extent of the impact that high production of insects might have is not clear [84]. A study by Lundy and Parrella [108] suggests that feeding bio-waste to insects could make insect production more environmentally feasible.

**Figure 1:**
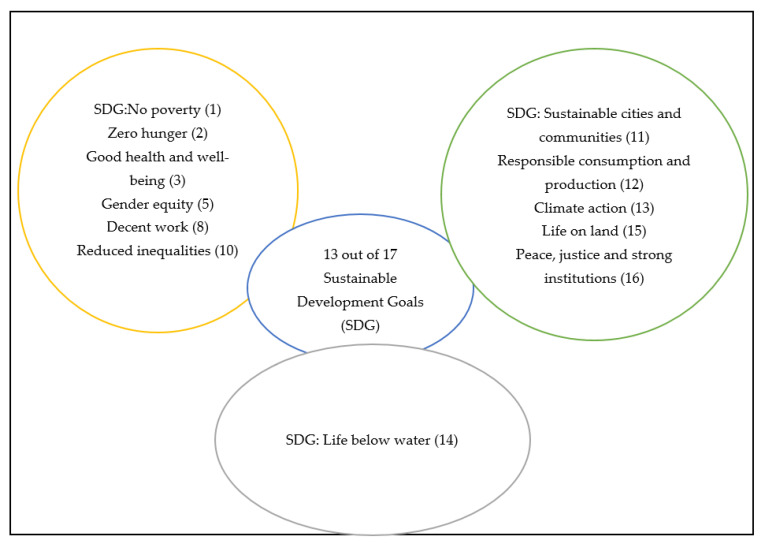
Schematic presentation of the 13 out of 17 SDGs aligned with edible insects.

According to Usman and Yusuf [25], using insects as food and feed can assist in achieving the sustainable development goals (SDG). Figure 1, below, shows the 13 SDGs that can support the achievement of these goals. The major advantages of insect farming, compared to livestock production, is the lower amount of greenhouse gas produced by insect farms (SDG 11, 13) and the use of less land and water (SDG 14, 15). Furthermore, fishmeal that originally comes from overexploitation of marine resources that is usually used in animal feed can also be reduced because insects will be used as a source of animal protein in poultry feeds (SDG 14, 12) [26]. Figure 1 summarises the importance of edible insects and their potential role in the attainment of the sustainable development goals (SDG). Since edible insects are associated with more ecological advantages, in comparison to traditional meat, they might be able to sustain the local environment by maintaining the diversity of habitats for other life forms, especially since they also require minimal land during their production stage [74]. In addition, capturing and consuming insects that are considered as pests to agricultural crops helps to reduce the use of pesticides in the agricultural sector (SDG 1, 2, 12) [78]. Kim [88] has reported that insects have the ability to address problems related to energy, water, land, food security, and to the traditional food supply chain.

Chia et al. [109] further reported that an inclusive business model that can include insects as ingredients in feed or food may contribute to improving the livelihoods of many communities, solving socio-economic and environmental problems in developing countries. These SDGs were officially adopted in January 2016 and the United Nations used them as a guideline to end all forms of poverty, fight inequalities, and tackle climate change. This will enable poor and middle-income and high-income communities to work together to achieve these goals. However, efforts to achieve the SDGs are felt differently among poor and rich people, women and men, developing or developed countries [109]. In order to achieve SDGs, inclusive business models should be developed and used to enable households, small and medium businesses to secure access to affordable goods and services, which are relevant to sustainable livelihoods.

Possible hazards from insect consumption are contaminants, for example, heavy metals, mycotoxins, pesticide residues, and pathogens [28]. The overuse of edible insects can also lead to their disappearance and extinction. For example, the overexploitation of the caterpillar, in Botswana and South Africa, has led to their disappearance in these countries [80,110] and, in Indonesia, weaver ants were depleted due to a lack of knowledge on the harvesting practices [111]. Halloran and Münke [112] have stated that the lack of all-inclusive legislation to govern the production and trade of insects is considered the greatest barrier to development in this sector. Dube et al. [113] have also reported a decrease in the frequency of traditional practices of entomophagy in developing countries and communities where insect consumption was once widespread. Van Huis et al. [28] proposed the merging of modern science with traditional knowledge and food culture, which would be invaluable to the development of innovations and increase mass-production and processing technologies of edible insects and, in turn, contribute to preventing the disappearance of wild populations. Even though health and safety issues have been mentioned, Tan et al. [114] have suggested that the incorporation of insects into food products will likely help to increase consumer acceptance, particularly in developed countries.

Food safety and quality control in Kenya is governed by multiple government agencies, which include the Department of Trade, Industrialisation, Public Health and Sanitation, and Livestock, Fisheries Development, and Agriculture [112]. In Thailand, the different types of insects that are used for human consumption, are authorised by the Ministry of Public Health and insect products are regulated by general food laws, such as the Food Act of 1979 [111]. In some countries, for example, Zambia and Zimbabwe, customary laws play an essential role in governing the production and harvesting of edible insects [39,115] and, in Europe, certain countries such as Belgium, the Netherlands and Switzerland have legislation regulations governing the production and marketing of edible insects [116]. The lack of laws governing the production, processing and marketing of edible insects in developing countries could disadvantage insect producers, especially their ability to access the export market and earn foreign currencies. Therefore, the development and implementation of coordinated food regulations for edible insects in developing countries are urgently needed [117]. Table 2 shows a list of certain countries that have some policies or legal framework that guides the use of edible insects.

It is important to emphasise the standardization and quality control aspects of edible insect farming. The lack of legislation governing the edible insect sector as part of agricultural activity is problematic and concerning. This creates challenges for insect rearing farmers with regard to compliance issues [118]. The legal framework governing insect farming will help to develop more systematic procedures for the mass production and processing of edible insects. [118]. These barriers will have an effect on using edible insects to attain sustainable development goals. It is important that each country develop its own legislative framework governing the edible insect sector, no one-size-fits-all approach will work because of different dynamics such as culture and business tradition [119]. The insect can only be a sustainable protein source of the future if current legal measures are set up. Furthermore, the production of insect-based protein using food by-products is two times more environmentally beneficial than conventional animal protein [120].

## 8. Interaction of Indigenous Knowledge and Developing Scientific Data

Integrating indigenous and scientific knowledge is gradually growing along various lines of argument. However, the practice of knowledge integration continues to face some challenges [122]. Therefore, it is important to assess the vulnerability of indigenous communities. In addition, how the collaboration between Western and indigenous knowledge can mutually benefit in a culturally compatible and sustainable manner [123]. Ghosh et al. [124] conducted a study in Benin on how people in two locations perceive entomophagy. It was reported that community members that were practising entomophagy are aware of safety issues concerning warning colouration that warns them of harmful and toxic insects in their communities. This knowledge can be useful to the scientific community that studies aposematic animals. Ghosh et al. [124] emphasised the importance of entomophagy in the two communities of Benin and further recommended that insect-rearing facilities should be established in those communities to preserve the culture of entomophagy, improve the livelihood and enhance the economic growth of other communities. This is clear evidence of collaboration between traditional and indigenous knowledge system where both partners mutually benefit from the exercise. Furthermore, Meyer-Rochow and Changkija [125] reported that the domestication of silkworm and the use of its product, silk, in Northeast India could have arisen from the practice of eating wild silkworm by the indigenous communities. Silk is now a multibillion-dollar industry and has been fully commercialised with around 150,000 tons of silk created every year [126,127]. Insect production is a very big industry that includes crop pollination, agricultural protection, health, pharmaceuticals, pet, implements for conduction of research, and livestock nutrition [127]. The practice of Muga culture in Northeast India is a clear example of the integration of indigenous knowledge system and traditional science. This indigenous technical knowledge (ITK) needs to be thoroughly understood, documented, and critically validated in different forms using modern techniques so as to integrate the effective ones into the farming system. This will help to reduce dependence on external inputs, increase sustainability, and reduce the cost of production. By incorporating ITK into the research and development agenda, production of Muga raw silk can be increased which in turn improves the livelihood and income of local communities [32].

## 9. Conclusions

Indigenous knowledge systems can be used to build resilience to climate change and increase the production of edible insects. Insect species, intended for human consumption, should be selected, managed, and prepared by considering the traditional knowledge acquired in countries where insect consumption is customary. African countries should develop legislative frameworks for the use of insects as food and feed, since this could facilitate the export/import market. Efforts to avoid overexploitation of edible insects from the wild will also contribute to the development and sustainability of the sector. This can be achieved by creating a sustainable production system that will safeguard the environment and ensure food safety and security. ITKs need to be thoroughly understood, documented, and critically validated. The knowledge acquired through ITKs can be incorporated into a research and development agenda that will ensure sustainability. Insect conservation should also be taken into consideration to avoid overexploitation.

## Figures and Tables

**Table 1:** Production and processing of common edible insects in Africa.

Common Name	Scientific Name	Production and Processing Method	Stage Consumed	Reference
Mopane worm	*Gonimbrasia belina*	Harvested from tree and ground prior to pupating; undigested material removed; boiled in salt water and then sun-dried	5th Instar stage	[37]
Field cricket	*Gryllus campestris*	Traditional method: digging them out from their habitat, then either fried or grilled	Adult	[82]
Edible stinkbug	*Encosternum delegorguei*	Insects are collected from tree branches in the morning, using long hooks or by climbing the trees and shaking them off branches and leaves; warm water is used for killing them and as a heating procedure before being stored.	Adult	[83]
Winged termite	*Macrotermes subhyalinus*	A light source and large bowl of water are used to trap them; best toasted or lightly fried until they are slightly crisp	Adult	[82]
Grasshopper	*Kraussaria angulifera*	Harvested from the wild; normally fried and grilled	Adult	[82]

**Table 2:** Common legislative bodies governing insects’ production and consumption in different countries.

Country	Legislative Agencies	Source
Kenya	Kenya Bureau of Standard (KEBS), Kenya Agricultural Research Institute (KARI), Kenya Plant Health Inspectorate Services (KEPHIS)	[121]
Thailand	Ministry of Public Health	[112]
Zambia	Customary laws	[115]
Zimbabwe	Customary laws	[39]
Belgium (crickets, locust, mealworms, moths, and silkworm)	Federal Agency for the safety of the food chain	[116]
Switzerland (crickets, locust, and mealworm)	Federal council	[116]
China (silkworm)	Ministry of Health	[116]
South Korea (crickets and mealworms)	Korean Food and Drug Administration	[116]

## Data Availability

Not applicable.
